# Genome Sequence of *Geobacillus stearothermophilus* DSM 458, an Antimicrobial-Producing Thermophilic Bacterium, Isolated from a Sugar Beet Factory

**DOI:** 10.1128/genomeA.01172-17

**Published:** 2017-10-26

**Authors:** Kevin Egan, Philip Kelleher, Des Field, Mary C. Rea, R. Paul Ross, Paul D. Cotter, Colin Hill

**Affiliations:** a School of Microbiology, University College Cork, Cork City, Ireland; b APC Microbiome Institute, University College Cork, Cork City, Ireland; c Teagasc Food Research Centre, Moorepark, Fermoy, Co. Cork, Ireland

## Abstract

This paper reports the full genome sequence of the antimicrobial-producing bacterium *Geobacillus stearothermophilus* DSM 458, isolated in a sugar beet factory in Austria. *In silico* analysis reveals the presence of a number of novel bacteriocin biosynthetic genes.

## GENOME ANNOUNCEMENT

*Geobacillus* spp. are thermophilic, Gram-positive, aerobic or facultative aerobic, spore-forming bacteria, and are highly resistant to heat when in spore form (1). The genus *Geobacillus* and its species have been shown in recent years to produce a number of bacteriocins or bacteriocin-like inhibitory substances (BLIS) (2–4). Here, we report the full genome sequence of *Geobacillus stearothermophilus* DSM 458, which was isolated from sugar beet juice obtained from extraction installations in Austria. It has been shown to produce a narrow-spectrum antimicrobial substance that targets other geobacilli, the basis of which requires further characterization (5). In order to identify the gene(s) responsible for the production of this antimicrobial substance, we completed full-genome sequencing of this bacterium, for which the 16S rRNA sequence had previously been determined (GenBank accession number AY608931).

Cells of *G. stearothermophilus* DSM 458 were grown to mid-log phase in brain heart infusion (BHI) broth and centrifuged at 5,000 rpm for 20 min. A 600-mg pellet of cells was then snap frozen by placing the centrifuge tube into ethanol which had been previously cooled to −80°C. Chromosomal DNA was isolated by commercial sequence provider GATC Biotech, Ltd. (Konstanz, Germany). Single-molecule real-time (SMRT) sequencing was performed on a Pacific Biosciences RS II sequencing platform (executed by GATC Biotech, Ltd., Germany) to a mean fold coverage of 147.88. *De novo* assembly of the genome was performed using the SMRTPortal analysis platform (version 2.3.1), utilizing the RS_HGAP_Assembly.2 protocol. This resulted in a single contiguous chromosome of 3,466,824 bp and a G+C content of 52.11%.

Following assembly of the genome, open reading frame (ORF) prediction was performed using the Prodigal v2.5 prediction software (6) and confirmed using BLASTx alignments (7). The genome was then automatically annotated using BLASTp (7) against the nonredundant protein database curated by the National Center for Biotechnology Information (NCBI). In addition to this, manual curation of the genome was verified using the Artemis genome browser and annotation tool (8), i.e., inspection of ORF results, adjustment of start codons where necessary, and identification of pseudogenes. Further genomic analysis utilized the programs tRNAscan-SE (9), RNAmmer (10), PHAST (11), and CRISPRFinder (12), showing that the genome contains 3,525 genes, 3,361 proteins, 43 pseudogenes, 32 rRNAs, 89 tRNAs, 4 putative phages, and 5 clustered regularly interspaced short palindromic repeat (CRISPR)-associated repeat regions. Additionally, BAGEL3 (13) predicts the presence of a novel circularin-like bacteriocin with a 46% identity to circularin A (14), as determined by pairwise alignment using BLASTp (7). AntiSMASH v4.0.2 (15) also highlighted the presence of this circularin-like bacteriocin and a gene cluster encoding a bacteriocin with homology to Linocin M18 (16). The Linocin M18-like bacteriocin was determined to have a 25% identity to the Linocin M18 protein sequence, as determined by pairwise alignment using BLASTp (7).

Combining the *in silico* analysis with *in vitro* experimentation should ultimately lead to characterization and greater understanding of the antimicrobial potential/nature of this strain. Furthermore, the availability of this genome will ultimately contribute to the accuracy of *in silico* prediction software for the genus of *Geobacillus* in the future.

### Accession number(s).

The full genome sequence of *Geobacillus stearothermophilus* DSM 458 has been deposited in GenBank under the accession number CP016552.
